# Macon Medical Society

**Published:** 1887-02

**Authors:** 


### THE MACON MEDICAL SOCIETY.

Stenographically Reported for The Atlanta Medical and Surgical Journal by C. G. Link.

Stated Meeting, December 7, 1886.

President E. G. Ferguson in the chair.

Dr. J. Emmett Blackshear read an essay on the

### MANAGEMENT OF THE THIRD STAGE OF LABOR.

Dr. Blackshear said: The object to be attained is the freeing of the uterus, in a reasonable length of time, of its remaining contents, the placenta and membranes. The danger of hemorrhage, septicæmia, etc., from undue retention of the secundines renders the management of this stage of labor a matter of great importance.

The first question that presents itself is as to whether interference is always necessary. Sometimes the placenta is detached and expelled with the child; at others it comes away with the second, third or fourth after-pain. Some obstetricians prefer, therefore, to wait for the placenta to become detached spontaneously unless severe hemorrhage should indicate earlier removal. To admit that interference, as a rule, is necessary would seem to imply that nature, while imposing upon women the duty of child-bearing, had imperfectly provided her with the power of performing the parturient function; but it must be remembered that civilization, in its devitalizing effects upon the race, has enfeebled many of the bodily functions, and this may be among them. It cannot be doubted that nature, in creating the progenitors of the race, performed her work in the highest degree of perfection, but a violation of her laws has entailed innumerable evils.

While the expectant method may do well in many cases, interference within proper limits can in nowise do harm, while it is often absolutely necessary. As a rule the detachment of the placenta, when left alone, takes place within an hour or two (often earlier) after the delivery of the child. I agree with Dr. Wesley Davis, of Worcester, Massachusetts, who, in an article published in the *American Journal of Obstetrics* for November, 1884, says: "The period immediately after the delivery of the child, before there has been time for the parts to recover themselves and take on tonic contraction, is above all others that most favorable for the easy expulsion of the secundines. The earlier this is accomplished the more thorough and perfect the contractions that can be secured and the less the loss by hemorrhage, and consequently a lessening of the tendency to absorption from without, whether septic or not." He delivers the placenta before ligating and severing the cord.

I am aware that it is not fashionable at the present day to advocate the procedure of making traction upon the cord, and yet, at the risk of being considered non-progressive, I unhesitatingly say that, in my opinion, it is admissible and proper. But it must be borne in mind that there is a right and a wrong way to proceed in the matter. The indiscriminate pulling at the cord with the view of removing the placenta is greatly to be deprecated.

The objection urged by some that there is danger of inverting the uterus is groundless when traction is properly made. The time (and the only time) to make it is during the uterine contractions. When then made it may be practiced with perfect impunity to any extent the cord will bear. Thus made it does not act upon the point of local insertion, but upon the placenta as a whole. It is not until it passes into the vagina, beyond the influence of the contracting uterus, that it becomes inverted.

My practice is, so soon as the child is delivered and given into the hands of the nurse, to apply massage, not confining myself to circular friction, as in the first stage of Crede's method, but by gentle squeezing, kneading, hand and finger pressure, etc., in addition. In this connection, Dr. Douglas Graham, in his admirable work on massage, says: "For fulfilling such important indications as arousing, equalizing and strengthening the contractions of this hollow, involuntary, muscular organ under such momentous circumstances, even though applied in a mediate manner, there is no longer any question of the well-known efficiency of kneading, squeezing and pressure." But it is not in the parturient stage of the uterus alone that massage is applied with most satisfactory results. To its virtue in attonic conditions of this organ generally, I am prepared fully to subscribe. Indeed, in the treatment of many of the ills of life it is one of the best of curative agents; and, in the language of the above-named author, "If it were as easily administered as pills, powders and liquids, its use would be much more extensive."

So soon as I find the womb contracting, I take hold of the cord and make traction downwards and backwards. If the placenta is not delivered with the first after-pain, I renew the massage until I find the uterus again contracting, when, as before, I make traction on the cord, and so continue the two processes until the placenta is removed. When these measures fail, I remove the placenta by the introduction of my hand into the uterus. I sometimes, and always when I know the woman to be of hemorrhagic diathesis, immediately on the expulsion of the placenta (or better perhaps of the child), administer a dose of ergot. When this stage of labor is perfectly performed, the membranes, as well as the placenta, are expelled in their entirety. To be sure that such is the case, their anatomical relations should be borne in mind. When any considerable portion of the membrane is found to have been left in the uterus, it may be removed by introducing the hand and taking it away, though the danger arising from such retention has doubtless been greatly exaggerated. It is better, however, that the uterus should be entirely cleared, as the interior of the organ will thus more speedily return to a healthy condition. I regard the binder as entirely useless and never apply them.

Dr. Holt said: As soon as the second stage of labor is completed, the child having cried vigorously, or the umbilical cord having ceased to pulsate, it is then severed, securely enveloped in suitable covering, and handed to the nurse. It is then my invariable custom to seat myself beside the patient, placing my left hand on the uterus to satisfy myself that it is firmly contracted. For a number of years I have usually (*except in a primipara*) at this juncture administered a drachm of Squib's fluid extract ergot, with a view of not only insuring permanent contraction of the womb, but to assist in expelling coagula, thereby lessening to a great extent the occurrence of after-pains, which in a great many women are more harassing and annoying than the pains of labor. Within the last few months much has been said and written by prominent obstetricians in various sections of the union condemning, or at least questioning, the propriety of the routine practice of administering ergot after the termination of the second stage of labor, claiming that it is wise to wait until there is some indication for its administration, others contending that it often produces contraction, thereby imprisoning the placenta within the womb. My experience has taught me that ergot given by the mouth will not produce its characteristic effects under fifteen or twenty minutes — here the danger of imprisoning the placenta is almost nil — that the only ill effect I have seen is sometimes nausea and vomiting, which at this particular juncture is, to say the least, uncomfortable to the patient. Personally I feel much more comfortable for my patient's safety as regards hemorrhage when I know she has a prompt dose of ergot, and until further experience, shall not deviate from my former custom. Keeping my hand on or above the uterus, watching closely its behavior, I confidently expect within twelve or fifteen minutes to feel a slight contraction or hardening of the womb; then I use Credé's method (which is familiar to all of you) to express the placenta. I would here remark that if the effort is not made *too soon* (and I find that I have rarely to wait more than fifteen minutes) that you will almost always be successful, it not only being driven out of the uterus, but often out of the vagina. When it is on the vagina I introduce my hand, twisting the membranes into a cord, so that they shall be removed *entire*. It is my custom to examine it after removal to see and know that none is left behind. If after examination a portion is wanting, I at once introduce one or two fingers and search for it till found. I rarely leave the bedside for thirty or forty minutes, occasionally placing my hand on the uterus to assure myself that it is still well contracted, it being an axiom in obstetrics that a well-contracted womb cannot bleed. My patient's condition being satisfactory to myself, I order the nurse to bathe the genitals thoroughly with warm water or carbolized water, if convenient (ʒi to Oj), superintend the removal of soiled linen, change her position from back to side, or *vice versa*, place a warm napkin slightly oiled under the genitals and direct her to be kept absolutely quiet, positively forbidding the officious visits of garrulous old women and inquisitive neighbors. My own opinion is that it is all-important that a woman, should she desire a good recovery, that she have an hour or more of sweet, refreshing sleep after the trying ordeal of labor. Should she be unable to get the desired sleep, unless there is some contra-indication, I direct an opiate given her, thereby insuring it. Occasionally, if my patient has had an easy labor and gives no evidences of fatigue, and is a multipara, I direct the child applied to the breast, which I feel assured will cause additional contraction of the womb, should the ergot have failed to produce the desired effect. Now coming to the vexed question of the parturient binder—much has and can be said *pro* and *con*. If my patient desires it (and some, believing that it preserves their maiden shape, are clamorous for its use), I consent to its application, but rarely advise its use unless the abdomen is unusually large and its walls very flaccid. I am willing to admit that it is a *support* to the abdominal walls; that it enables the patient to turn in bed with much more ease and comfort. *Per contra*, it is difficult, if not impossible, to keep them in position (and I have tried various shapes and patterns, to all of which there is the same objection) and in the summer months they are insupportably hot and uncomfortable. I direct the genitals kept *scrupulously clean*. Do not, as a general rule, direct antiseptic vaginal washes *except after* instrumental labors, or the lochia are offensive.

Dr. K. P. Moore stated: It has been my custom for a long while, just at the last two or three throes of labor, to administer a dose of ergot, and as a matter of precaution, patient taking several minutes to get benefit of ergot, I try to anticipate the last few efforts and expulsion of the fœtus, and give a dose of ergot just before the child is born, hoping thereby to give it in time in anticipation of any risk of post-partum hemorrhage. I am a strong believer in ergot and have frequently seen as decided benefit derived from it as from any other medicine. It has been my rule, ordinarily, to wait fifteen or twenty minutes before expelling the placenta, and if I cannot in due time with manipulation excite sufficient action on the part of the uterus, I introduce my hand and have never as yet seen any bad effects from it. I also question very much that any harm can be done by delivering the placenta immediately after taking child and handing over to the nurse. It might be well just here to speak of the precautions necessary in introducing hand for placenta or any other purpose. I plead guilty to not having always used the proper precautions myself, and can recall instances where I have delivered a child, with manipulation, getting my hands stained with blood, it afterwards becoming necessary to introduce my hand into the uterus, did so without washing my hands, and it is possible I may have produced a septic condition. I feel that should I again be called upon to deliver a placenta by the introduction of my hand I shall certainly be more cautious.

Dr. Brown said: There are three stages in a natural labor. Of these the third is the most important, and requires the most care in its management. Strange to say, the books give detailed instructions for conducting the second stage to a successful termination, but dismiss the first and third stages with half a dozen sentences. As a general thing the practitioner considers himself so busy and his time so valuable that he considers that he has done his duty to the patient when the placenta has been delivered, so washes his hands and dismisses the case. This is a most deplorable error, as this is the most dangerous part in the process of labor and requires a skilled physician.

I will first state what constitutes the three stages of labor. The first stage dates from the time of impregnation. I do not mean by this that the woman is in actual labor during this whole period, but that it is necessary that she should be under the observation of her family physician during the course of pregnancy. She is liable to have albuminuria and other concomitants, which will prove troublesome and probably fatal if not given proper prophylaxis. The second stage dates from the engagement of the head to the birth of the child. The third stage, which is the subject of our discussion this evening, dates from the birth of the child to the complete emptying of the uterus, which may be accomplished in half an hour or it may be several hours before it is complete. The first procedure in the management of this stage is to cut and ligate the cord. The cord should be ligated at two points after pulsations have ceased. The first should be tied two inches from the abdomen of the child, the second one inch from the former on the maternal end of the cord. This second ligation should be made to prevent hemorrhage through the vessels of the cord, which sometimes occurs when the placenta is adherent and proves exhausting to the mother. Then there may be twins and the circulation may be directed from the placenta, which is often used in common by both children; hence the remaining child suffers from the loss of blood flowing through the other cord. Two years ago I had a case in this city in which the mother gave birth to triplets; all did well for two days when one died; the other two lived to be six months old and died of cholera infantum. Two of these children were nourished from one placenta; the third had one to itself. I believe that preventing the escape of blood from the cord assists in detaching the placenta. I am opposed to giving ergot to cause the expulsion of the placenta. I have had two cases in which it produced hour-glass contraction, enclosing the placenta firmly into the fundus of the uterus. Nothing could be done until I had thoroughly anæsthetized the patient, causing relaxation of the uterine tissues. If the patient is much exhausted I give a stiff drink of whisky or brandy and wait a while on the placenta—that is, if there is not much hemorrhage to make delay dangerous. If there is much hemorrhage I proceed at once to expel the placenta by Credé's method, with the exception of traction on the cord, which I consider hazardous. When the placenta has been delivered, I administer a drachm of the fluid extract of ergot (Squibb's preferred). I then knead the uterus with one hand and follow it down with the other pressed firmly down into the abdominal walls above the fundus of the womb, using gentle but firm pressure. This should be continued for at least half an hour, or until all clots have escaped from the womb and the organ has contracted to the size of an ordinary orange. This thorough contraction of the womb is a valuable preventive of after-pains which are usually so annoying to the woman in convalescence. After-pains are caused by the efforts of the womb to get rid of clots left in during confinement. Now, then, I shall state my last procedure in managing this stage before turning the patient over to the nurse for a few hours. This is the application of a bandage on the woman; this is not put on for the same purpose for which it is used by old grannies, viz., "to preserve the woman's form." But I use it for the purpose of getting a thorough contraction of the uterus—that is, to complete the process which I began with my hand. I use a bandage which is an invention of my own, and it has given entire satisfaction in every case in which I have used it. My patients express themselves as having derived comfort from the use of the bandage; hence I shall continue its use as long as it proves satisfactory. The bandage consists of a strong piece of domestic, which must be long enough to meet around the patient's body and hips. Then it must extend from about the middle of the thighs to just below the breasts. The patient is directed to lie at full length with the thighs together. After placing over the vulva a pad, or light and porous compress to absorb the discharges, I proceed to fasten the bandage. I begin by drawing the bandage tight and pinning from below up the whole extent to the breasts. I sometimes place a pad just above the fundus of the womb, under the bandage, which keeps the womb firmly pressed downward and excites tonic contraction. I sometimes have the bandages ready made and use cords and eyelets like those in a corset. I let bandage remain twelve or twenty-four hours, or more if it is necessary to continue the contractions. After-treatment is same as used in cases of confinement.

Dr. McHatton stated: When I first studied medicine I invariably gave a dose of ergot as soon as the position of the head was such that the forceps could be applied, if necessary; from that I drifted into giving ergot after the head was delivered, and now never give it until the child is delivered and frequently not at all. My usual procedure in the third stage is to take my seat by the bedside of the patient, place my hand on the uterus and keep it there as long as I feel it to be of ordinary size; I do not bother it, but simply let my hand lie on the abdomen. As a rule I get the placenta in the first, second or third pain. If I do not get the desired contraction after the placenta is delivered, and see an inclination to expand again, I give a dose of ergot. Regarding bandages, like Dr. Holt, if the patient so desires, I use them, but prefer not for the reason that I think the preserving of the figure depends upon the length of time she stays in bed more than anything else. I always carry a bottle of carbolic acid in my little case, and if I have to deliver placenta by introduction of hand, wash my hands thoroughly in a solution of carbolic acid In regard to delivery of placenta, I think that the longest case I have had was forty minutes. I was sometime ago called in consultation with Dr. Brown on a case where ergot had been given, and after the child had been delivered, there was an absolute contraction of the uterus. It was impossible to introduce your fingers, and the condition of the patient was such that I advised Dr. Brown to sit by the bed and watch the uterus, I being compelled to be away for perhaps three hours. When I came back the same condition of affairs existed, little or no bleeding, but after a few minutes the blood commenced to flow. I am firmly convinced that the contraction was caused by the ergot, and also that she was the better off for being let alone than by having a forcible delivery while in that condition.

Mrs. Dr. Fuller stated: In regard to contraction, my preceptor believed in massage, and I have followed in his footsteps. I usually take my place by the bedside as soon as the child is delivered, put one hand upon the uterus, until I feel contraction, to assist in bringing the placenta away, and thus far I have had no trouble. In regard to the use of ergot I never give it unless I see some decided need of it. In the second stage, when contractions are slow and do not continue long enough to be of any use, to increase the pain I give a few drops of ergot. In a case, yesterday morning, I gave thirty drops in three-quarters of an hour, and gave thirty more from eight in the evening to four this morning, and I found that the pains were slight; I proceeded in this way, giving no ergot after the child was born, it being only one hour after the last was given before it was born. Everything passed off all right after giving ergot, and I had no trouble about placenta.

Dr. E. C. Ferguson stated: From what I have seen of the use of ergot, I have come to the conclusion that it is perfectly worthless, nor do I believe in making traction on the funis for fear of breaking it or inverting the uterus, but prefer delivering the placenta by introducing my hand and withdrawing by grasping the free border. I find that nothing is equal to ice in case of post-partum hemorrhage, and, after trying the bandages, I find that they are no good at all.

---

INTERNATIONAL MEDICAL CONGRESS, VACANCIES FILLED.—Dr. J. J. Chisolm, of Baltimore, Maryland, has been appointed President of the Section of Ophthalmology, in place of Dr. E. Williams, who was compelled to resign on account of ill health. Judson B. Andrews, M. D., Superintendent of the Hospital for the Insane at Buffalo, New York, has been appointed to the place made vacant by the death of Dr. John P. Gray, as President of the Section of Psychological Medicine and Nervous Diseases. No vacancies now remain in the list of the chief officers of the preliminary organization of the Congress or its Sections, and our information from all departments is of the most encouraging character.—*Journal of the American Medical Association.*

